# Investigation of Factors Causing Nonuniformity in Luminescence Lifetime of Fast-Responding Pressure-Sensitive Paints

**DOI:** 10.3390/s21186076

**Published:** 2021-09-10

**Authors:** Yasuhiro Egami, Yuya Yamazaki, Naoto Hori, Yosuke Sugioka, Kazuyuki Nakakita

**Affiliations:** 1 Department of Mechanical Engineering, Aichi Institute of Technology, 1247 Yachigusa, Yakusa-cho, Toyota 470-0392, Aichi, Japan; yuuuya929@gmail.com (Y.Y.); hori.naoto.gk1.12.6@gmail.com (N.H.); 2 Aeronautical Technology Directorate, Japan Aerospace Exploration Agency, 7-44-1 Jindaiji Higashi-machi, Chofu-shi, Tokyo 182-8522, Japan; sugioka.yosuke@jaxa.jp (Y.S.); nakakita.kazuyuki@jaxa.jp (K.N.)

**Keywords:** pressure-sensitive paint, fast-responding, luminescence lifetime, uniformity

## Abstract

Factors that cause nonuniformity in the luminescence lifetime of pressure-sensitive paints (PSPs) were investigated. The lifetime imaging method of PSP does not theoretically require wind-off reference images. Therefore, it can improve measurement accuracy because it can eliminate errors caused by the deformation or movement of the model during the measurement. However, it is reported that the luminescence lifetime of PSP is not uniform on the model, even under uniform conditions of pressure and temperature. Therefore, reference images are used to compensate for the nonuniformity of the luminescence lifetime, which significantly diminishes the advantages of the lifetime imaging method. In particular, fast-responding PSPs show considerable variation in luminescence lifetime compared to conventional polymer-based PSPs. Therefore, this study investigated and discussed the factors causing the nonuniformity of the luminescence lifetime, such as the luminophore solvent, luminophore concentrations, binder thickness, and spraying conditions. The results obtained suggest that the nonuniformity of the luminophore distribution in the binder caused by the various factors mentioned above during the coating process is closely related to the nonuniformity of the luminescence lifetime. For example, when the thickness of the binder became thinner than 8 μm, the fast-responding PSPs showed a tendency to vary significantly in the luminescence lifetime. In addition, it was found that the luminescence lifetime of fast-responding PSP could be changed in the depth direction of the binder depending on the coating conditions. Therefore, it is important to distribute the luminophore uniformly in the binder layer to create PSPs with a more uniform luminescence lifetime distribution.

## 1. Introduction

A pressure-sensitive paint (PSP) is an optical pressure sensor capable of capturing the pressure distribution on a model surface with high spatial resolution [1]. A sprayable fast-responding PSP (fast-PSP), such as polymer/ceramic PSP (PC-PSP), has been developed for the time-resolved measurement of pressure fluctuation [1,2,3,4,5]. Unsteady PSP measurements have been conducted using both intensity and lifetime methods. The lifetime method measures the change in the luminescence lifetime of PSP with pressure.

The most significant advantage of the lifetime method is that the pressure distribution can be measured only from the “wind-on” data taken when the wind tunnel is turned on [1,6]. A lifetime imaging method measures pressure from the ratio of the two “wind-on” images obtained at different gate timing, while the intensity method requires both “wind-on” and “wind-off” images taken when the wind tunnel is turned on and off, respectively. By eliminating the “wind-off” images, we can reduce the measurement errors caused by the deformation or movement of the model and changes in the distribution of excitation light and temperature on the model between the “wind-on” and “wind-off” conditions. Much effort has been made to develop the lifetime imaging method [6,7,8,9,10,11,12,13,14,15,16,17]. However, it is known that the luminescence lifetime of PSP is not spatially uniform, even under uniform pressure and temperature conditions [12,13,15,16,17]. Sugioka et al. reported that a polymer-based PSP applied to a real wing showed considerable spatial variation in the luminescence lifetime [13]. Therefore, the ratio images obtained under the known reference conditions, which usually refers to the “wind-off” condition, are additionally employed to compensate for the spatial nonuniformity of the lifetime. However, the use of both “wind-on” and “wind-off” images dramatically diminishes the lifetime imaging method’s advantages. In particular, fast-PSPs, which use porous and heterogeneous binders, show considerable variation in luminescence lifetime compared to conventional homogeneous polymer-based PSPs. However, the apparent reason for the nonuniformity of the lifetime remains unclear. In this study, two-typical fast-PSPs, one-component [2,5,18,19] and two-component [3,4,5,20,21,22,23] fast-PSPs, were investigated to determine the factors causing the nonuniformity of the luminescence lifetime. The effects of the luminophore solvents, the amount of luminophore applied, binder thickness, and spraying conditions on luminescence lifetime variation were investigated and discussed. By finding the conditions to create fast-PSPs with a more uniform emission lifetime distribution, we can improve the measurement accuracy of the lifetime imaging method using fast-PSPs. Furthermore, it is expected that a highly accurate lifetime imaging method can be realized without using reference images.

## 2. Lifetime Imaging Method and PSP Characteristics

**Figure 1:**
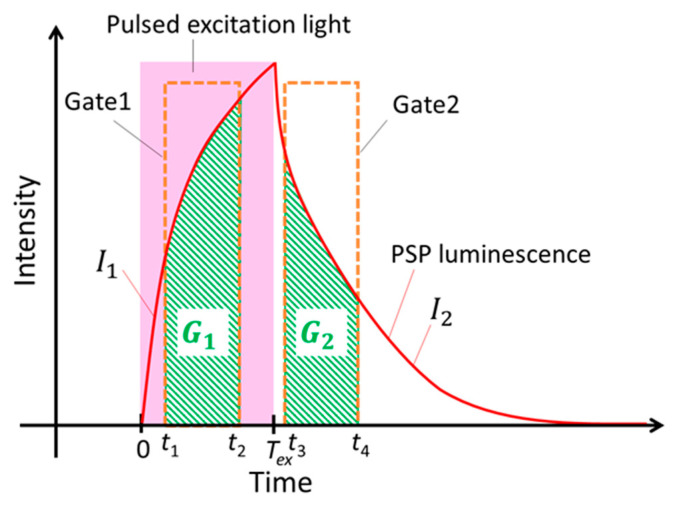
Schematic view of lifetime imaging method of PSP.

The luminescence lifetime change by pressure is, in addition to the change in luminescence intensity, represented by the Stern–Volmer relationship [1], as follows:

(1) $$\frac{\tau_{\mathrm{ref}}}{\tau }=A_{0}\left(T\right)+A_{1}\left(T\right)\frac{p}{p_{\mathrm{ref}}},$$

where τ and *p* are luminescence lifetime and pressure, respectively, and the subscript “ref” denotes a reference condition. The constants $A_{0}\left(T\right)$ and $A_{1}\left(T\right)$ are so-called Stern–Volmer constants and are a function of temperature *T*. Figure 1 shows the schematic view of the change in the luminescence intensity of PSP. Theoretically, the response of the luminescence intensity *I* of PSP to an excitation light E(t) can be described as a first-order system.

(2) $$\frac{dI}{dt}=-\frac{I}{\tau }+E\left(t\right),$$

where τ and *t* are the luminescence lifetime and time [1,6]. When the initial condition is I(0)=0, the solution to Equation (2) is given by Equation (3).

(3) $$I\left(t\right)=\int_{0}^{\;t}E\left(u\right)\exp\left(-\frac{\left(t-u\right)}{\tau }\right)du.$$

When PSPs are excited by pulsed light of width *T_ex_* from *t* = 0 to *T_ex_* in a micro-heterogeneous polymer matrix, they often exhibit multiple exponential decays as described by Equation (4).

(4) $$I\left(t\right)=\int_{0}^{\;t}E\left(u\right)\overset{n}{\underset{i=1}{\sum }}\alpha_{i}\exp\left(-\frac{\left(t-u\right)}{\tau_{i}}\right)du,$$

where $\alpha_{i}$ represents the weighting constants for $\tau_{i}\;(\alpha_{1}+\alpha_{2}+\ldots +\alpha_{n}=1$).

When the excitation light *E*(*t*) is a square wave, the luminescence intensity $I_{1}$ during excitation $\left(0<t<T_{ex}\right)$ and $I_{2}$ after excitation $\left(t>T_{ex}\right)$ is given by Equations (5) and (6).

(5) $$I_{1}\left(t\right)=I_{0}\int_{0}^{t}\overset{n}{\underset{i=1}{\sum }}\alpha_{i}\exp\left(-\frac{\left(t-u\right)}{\tau_{i}}\right)du=I_{0}.$$

(6) $$I_{2}\left(t\right)=I_{0}\int_{0}^{T_{\mathrm{ex}}}\overset{n}{\underset{i=1}{\sum }}\alpha_{i}\exp\left(-\frac{\left(t-u\right)}{\tau_{i}}\right)du=I_{0}\sum_{i=1}^{n}\alpha_{i}\tau_{i}\left(\exp\left(-\frac{t-T_{ex}}{\tau_{i}}\right)-\exp\left(-\frac{t}{\tau_{i}}\right)\right).$$

In this lifetime imaging measurement of PSP, two images are captured during the excitation and decay, as shown in Figure 1. The luminescence intensity of $G_{1}$ and $G_{2}$ is obtained by integrating $I_{1}$ and $I_{2}$ from $t_{1}\;$ to $t_{2}$ and from $t_{3}\;$ to $t_{4}$, respectively.

(7) $$G_{1}=\int_{t1}^{t2}I_{1}\left(t\right)dt=I_{0}\overset{n}{\underset{i=1}{\sum }}\alpha_{i}\left(\tau_{i}\left(t_{2}-t_{1}\right)+\tau_{i}{}^{2}\left(\exp\left(-\frac{t_{2}}{\tau_{i}}\right)-\exp\left(-\frac{t_{1}}{\tau_{i}}\right)\right)\right).$$

(8) $$G_{2}=\int_{t3}^{t4}I_{2}\left(t\right)dt=I_{0}\overset{n}{\underset{i=1}{\sum }}\alpha_{i}\tau_{i}^{2}\left(-\exp\left(-\frac{t_{4}-T_{ex}}{\tau_{i}}\right)+\exp\left(-\frac{t_{3}-T_{ex}}{\tau_{i}}\right)+\exp\left(-\frac{t_{4}}{\tau_{i}}\right)-\exp\left(-\frac{t_{3}}{\tau_{i}}\right)\right).$$

The ratio $R_{12}$ between $G_{1}$ and $G_{2}$ is given by Equation (9).

(9) $$R_{12}=\frac{G_{1}}{G_{2}}=\frac{\sum_{i=1}^{n}\alpha_{i}\left(\tau_{i}\left(t_{2}-t_{1}\right)+\tau_{i}{}^{2}\left(\exp\left(-\frac{t_{2}}{\tau_{i}}\right)-\exp\left(-\frac{t_{1}}{\tau_{i}}\right)\right)\right)}{\sum_{i=1}^{n}\alpha_{i}\tau_{i}^{2}\left(-\exp\left(-\frac{t_{4}-T_{ex}}{\tau_{i}}\right)+\exp\left(-\frac{t_{3}-T_{ex}}{\tau_{i}}\right)+\exp\left(-\frac{t_{4}}{\tau_{i}}\right)-\exp\left(-\frac{t_{3}}{\tau_{i}}\right)\right)}.$$

Since $R_{12}$ is a function of τ, it can be related to the pressure *p.* The relation between *p* and $R_{12}$ is determined in a calibration test.

## 3. Experimental Methods

### 3.1. Materials and Preparation

Fast-PSPs are composed of a pressure-sensitive luminophore, a polymer, particles, and solvents. There are two major types of fast-PSPs: one-component fast-PSPs (1C-PSPs) and two-component fast-PSPs (2C-PSPs). 1C-PSPs are prepared by mixing the luminophore, polymers, and particles in a single solvent and coating them together. In the other type, 2C-PSPs, the solution of polymer and particles is first applied to form a binder layer, and then the luminophore solution is applied onto the binder layer. While the first developed 1C-PSP had a slow time response of more than 250 µs, the 2C-PSP achieves a faster time response because the luminophores are distributed only near the surface [3,4,5,20,21,22,23].

In recent years, 1C-PSP has achieved a time response of about 10 µs, comparable to that of 2C-PSP, by reducing the size of the mixed particles from 250 nm to 30 nm and increasing the specific surface area [18,19]. Therefore, we employed 1C- and 2C-PSPs with different particle sizes in this study. In addition, a conventional polymer-based PSP without particles was also used for comparison.

Here, platinum tetrakis(pentafluorophenyl) porphyrin (PtTFPP, Porphyrin-Laboratories GmbH, Scharbeutz, Germany) was used as a pressure-sensitive luminophore in this study. As the polymer and particles, an ester polymer and hydrophilically treated titanium dioxide (TiO_2_) with average particle diameters *d* of 250, 30, and 15 nm (Tayca Corp., Osaka, Japan) were employed. The ester polymer and TiO_2_ were mixed at a particle mass content of 93 wt.%. Here, we define the particle mass content as the ratio by weight of the particles to the total weight of the particles and polymer.

#### 3.1.1. One-Component Fast-PSP (1C-PSP)

**Figure 2:**
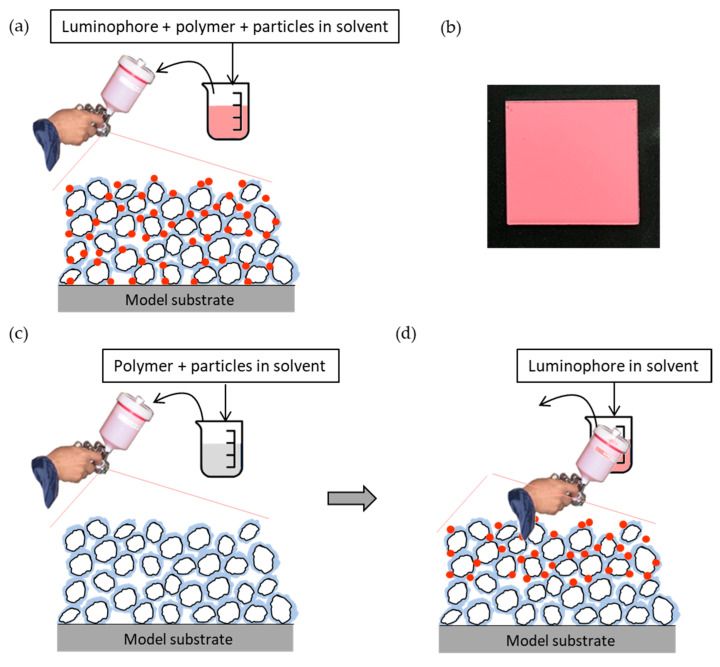
Spraying procedures for fast-PSPs and prepared sample: (**a**) 1C-PSP and (**b**) prepared 1C-PSP sample, and (**c**,**d**) 2C-PSP: In 1C-PSP, the luminophore, polymer, and particles are applied together to the model surface (**a**); in 2C-PSP, the polymer and particles, which are the binder material, are first applied (**c**), and then the luminophore solution is separately overcoated on the binder (**d**).

In the 1C-PSP, the luminophore and the binder materials were mixed together, as shown in Figure 2a. PtTFPP, TiO_2,_ and the ester polymer were mixed at the ratio of *x* mg:93 mg:7 mg in 1 mL of toluene (FUJIFILM Wako Pure Chemical Corp., Osaka, Japan). The luminophore amount of *x* mg per 1 mL of toluene was varied in the range of 0.5 to 4 mg/mL (0.43 to 3.42 mol/L). The 1C-PSP solution was stirred well using a magnetic stirrer and then sonicated to disperse TiO_2_ in the solution. The 1C-PSP solution was sprayed onto a 15 × 15 mm^2^ aluminum substrate using a spray gun (Minijet 3000 B HVLP, SATA GmbH, Kornwestheim, Germany). The binder thickness was varied from 2.2 to 15.7 μm at a PtTFPP concentration of 1 mg/mL. The prepared samples were dried under vacuum conditions in a chamber for 8 h before the test. Figure 2b is an example of a sample coupon of 1C-PSP created.

#### 3.1.2. Two-Component Fast-PSP (2C-PSP)

The ester polymer and TiO_2_ were dissolved/dispersed in toluene. The binder solution was stirred and then sonicated to disperse TiO_2_. The luminophore solution was prepared by dissolving PtTFPP in a 2:8 mixture of toluene and methanol (FUJIFILM Wako Pure Chemical Corp., Osaka, Japan) at a luminophore concentration of 1 mg/mL (0.86 mol/L). To investigate the effect of luminophore solvent on the luminescence lifetime, we also prepared samples with a mixture of toluene and methanol in the ratios (a) 10:0, (b) 6:4, and (c) 2:8. The luminophore concentration was also varied from 0.5 to 4 mg/mL (0.43 to 3.42 mol/L). The spraying procedure of 2C-PSP is shown in Figure 2c. The binder solution was sprayed onto the aluminum sample substrate using the spray gun (Figure 2c). The binder layer thickness was varied from 0.8 to 15.8 μm to evaluate the effect of layer thickness on the luminescence lifetime of fast-PSP. The luminophore (PtTFPP) solution was sprayed onto the precoated binder (Figure 2d). The prepared samples were dried under vacuum conditions in a chamber for 8 h before the test.

#### 3.1.3. Polymer-Based PSP

The polymer-based PSP was prepared by mixing PtTFPP and the ester polymer at 1 mg and 1000 mg per 1 mL of toluene. Then, it was applied to the aluminum sample substrate in the thickness range of 4–18.7 μm. After coating, the prepared samples were dried in a vacuum chamber as fast-PSPs above.

### 3.2. Luminescence Lifetime Measurement

**Figure 3:**
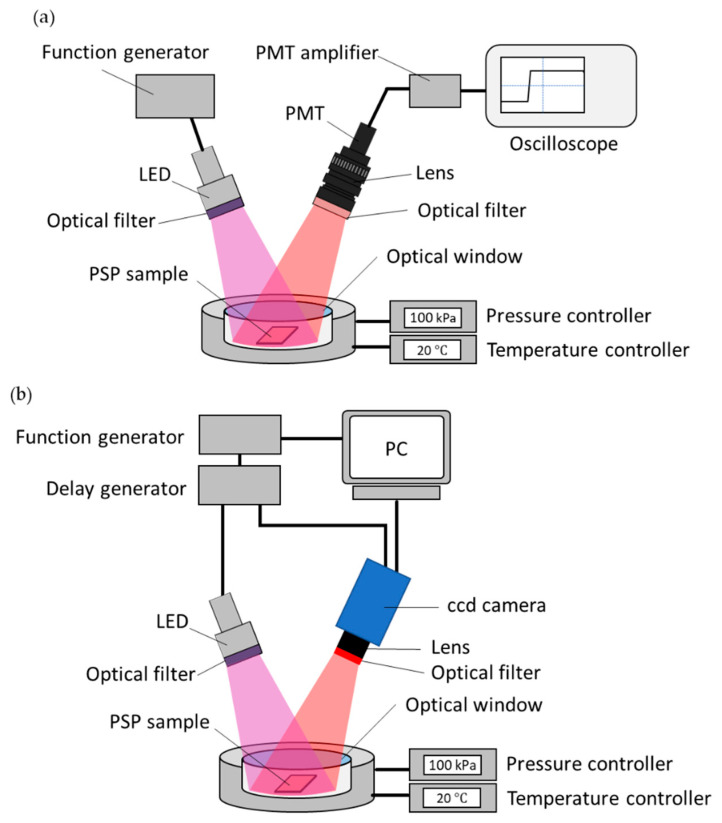
Schematic of the experimental setup for (**a**) luminescence lifetime measurement with a PMT, and (**b**) lifetime imaging with a CCD camera.

The luminescence lifetime of fast-PSPs was measured using a photo-multiplier tube (PMT). Figure 3a shows a schematic diagram of the lifetime measurement system using the PMT. The prepared PSP samples were placed on a stage in a pressure chamber. By using a pressure controller (PACE5000, Baker Hughes, Houston, TX, USA) and a temperature controller (TDC-1020A, Cell System, Yokohama, Japan), the pressure and temperature in the chamber could be controlled within the range of 1 to 200 kPa and 0 to 50 °C, respectively. PSPs in the chamber were excited with a 390 nm LED (IL-106UV LED, HARDsoft Microprocessor Systems, Kraków, Poland) using an optical lowpass filter of 490 nm (VIS 490, Asahi Spectra, Tokyo, Japan) and a heat absorption filter (HAF-50S-30H, Sigma Koki, Hidaka, Japan). The LED was operated in pulse mode with a function generator (33500B, Agilent Technologies, Santa Clara, CA, USA). The luminescence from PSP was captured by a PMT (H10721-1, Hamamatsu Photonics, Hamamatsu, Japan) with a camera lens (Ai AF NIKKOR 50 mm, Nikon, Tokyo, Japan) and a bandpass filter of 650 ± 20 nm (PB0650-40, Asahi Spectra, Tokyo, Japan). The obtained signal was recorded using a 10 bit oscilloscope (RTB2004, Rohde & Schwarz, Munich, Germany) through an amplifier unit of bandwidth from DC to 1 MHz (C12419, Hamamatsu Photonics, Hamamatsu, Japan). The luminescence lifetime was calculated by fitting the obtained decay curve of the luminescence intensity (*I*_2_ in Figure 1) using Equation (6). In this study, the pulse width *T* of excitation light was set to 10 μs. The averaged luminescence lifetime τ of PSP with multiple lifetimes can be expressed as shown in Equation (10).

(10) $$\langle \tau \rangle =\frac{\sum_{i=1}^{n}\alpha_{i}\tau_{i}^{2}}{\sum_{i=1}^{n}\alpha_{i}\tau_{i}}.$$

In this paper, *n* = 3 was used to evaluate the obtained results.

The spatial uniformity of the luminescence lifetime of fast-PSP samples was evaluated using a CCD camera (pco.2000, PCO AG, Kelheim, Germany). Figure 3b exhibits a schematic view of lifetime imaging using the CCD camera. Two images of *G*_1_ and *G*_2_ in Figure 1 were taken by synchronizing the 390 nm LED and the CCD camera with the TTL signal from LabVIEW^®^ through the function generator and a delay generator (DG535, Stanford Research Systems, Sunnyvale, CA, USA). In this study, the pulse width *T* of excitation light shown in Figure 1 was also set to 10 μs. Furthermore, *t*_1_ and *t*_2_ for gate 1 were set to 0 and 9 μs, and *t*_3_ and *t*_4_ for gate 2 were set to 11 and 25 μs, respectively. The ratio $R_{12}$ of $G_{1}\;$ and $G_{2}$ in Equation (9) was processed in MATLAB. The relation between $R_{12}$ and 〈τ〉 obtained from the calibration test was expressed as shown in Equation (11).

(11) $$\frac{{\langle \tau \rangle }_{\mathrm{ref}}}{\langle \tau \rangle }=B_{0}+B_{1}\frac{R_{12}}{R_{12}{}_{\mathrm{ref}}},$$

where the subscript “ref” denotes a reference condition, and $B_{0}$ and $B_{1}\;$ are constants determined by the calibration test.

## 4. Results and Discussion

### 4.1. Effect of Luminophore Solvents on 2C-PSP

2C-PSPs have been employed as a conventional sprayable fast-PSP [3,4,5,20,21,22,23]. The luminophore solution is applied over the precoated binder layer [3,4,5], as shown in Figure 2a.

By distributing the luminophore only near the binder surface, the responsiveness of the fast-PSP can be improved. Sugioka et al. [21,22] developed 2C-PSPs using an ester polymer that dissolves in organic solvents, such as toluene. However, when a luminophore solution of PtTFPP dissolved in toluene was sprayed on the binder layer, the 2C-PSP showed a slightly slower response time of 250 μs [21,23]. This slower response is because the toluene in the luminophore solution dissolved the polymer in the binder layer and changed the porous structure of the binder [20,21]. Instead of toluene, methanol, which does not dissolve the polymer, or a mixture of toluene and methanol in a ratio of 20:80 was used as a luminophore solvent; they could successfully improve the response time to 10–100 μs [22,23]. Here, three 2C-PSPs were prepared with toluene and methanol in the ratios of (a) 100:0, (b) 60:40, and (c) 20:80 (see Table 1). The 2C-PSPs were prepared by applying the above luminophore solutions onto the top of the precoated porous binder using TiO_2_ with a particle size of *d* = 250 nm. Toluene has a faster volatility rate and higher solubility of PtTFPP than methanol.

**Figure 4:**
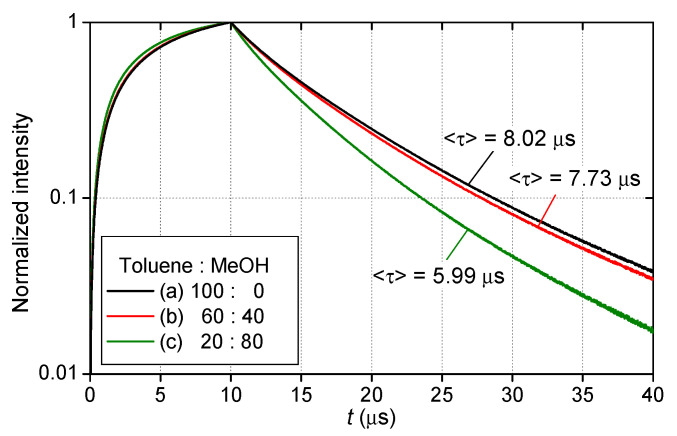
Luminescence waveforms during excitation and decay processes of 2C-PSP (*d* = 250 nm) prepared with toluene and methanol in the ratios of (a) 100:0, (b) 60:40, and (c) 20:80.

**Figure 5:**
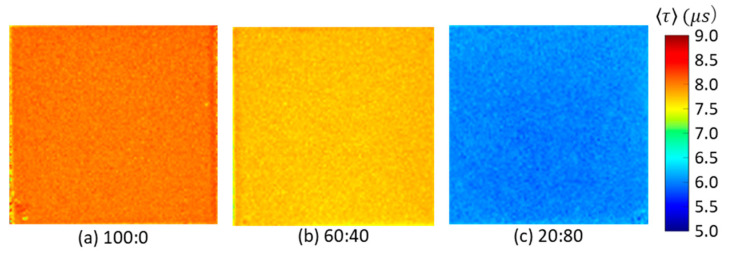
Distribution of luminescence lifetime 〈τ〉 of 2C-PSP (*d* = 250 nm) prepared with toluene and methanol in the ratios of (**a**) 100:0, (**b**) 60:40, and (**c**) 20:80.

Figure 4 shows the changes in the normalized luminescence intensity of the three 2C-PSPs during the excitation and decay process measured with the PMT in constant conditions of 100 kPa and 20 °C. Figure 5 presents the images of the luminescence lifetime measured by the CCD camera obtained in the same conditions as Figure 4. When the ratio of toluene in the mixed solvent was reduced, the 2C-PSP exhibited lower luminescence intensity and a longer luminescence lifetime. As the toluene percentage decreased from 100% to 20%, the averaged luminescence lifetime 〈τ〉 decreased from 8.02 µs to 5.99 µs as shown in Table 1; this corresponds to a 25% decrease in 〈τ〉. The difference in the solvent’s volatility and solubility of PtTFPP may have affected the distribution of the luminophore in the binder, resulting in differences in the luminescence intensity and lifetime.

**Figure 6:**
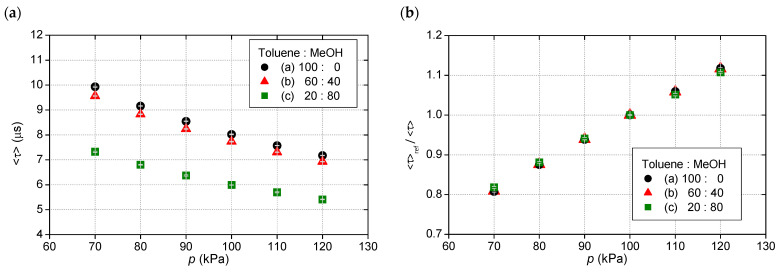
Pressure calibration of 2C-PSPs with different luminophore solvents: (**a**) Variation of 〈τ〉 by pressure and (**b**) Stern-Volmer curves.

**Figure 7:**
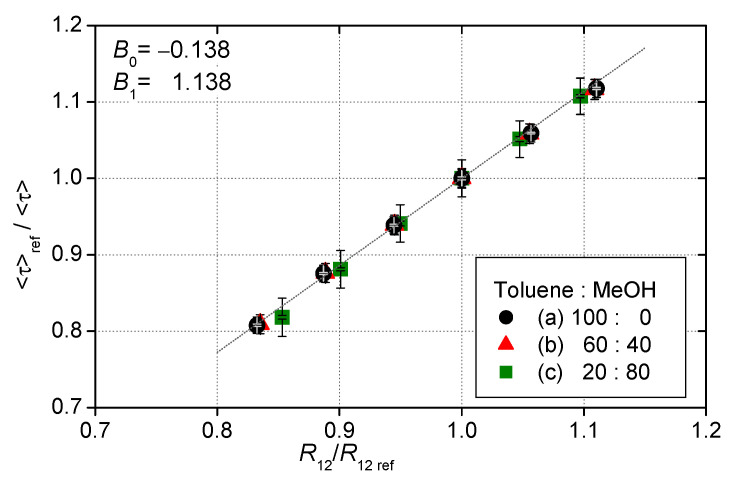
Relationship between $R_{12}\;$ and 〈τ〉.

Figure 6 shows the pressure calibration of these three 2C-PSPs. The 2C-PSPs had different luminescence lifetime depending on the solvents but almost the same pressure sensitivity of about 0.6%/kPa. The 1C-PSPs described below also showed nearly the same pressure sensitivity. In other words, an error of 1% in a luminescence lifetime at atmospheric pressure corresponded to a pressure error of approximately 1.6 kPa. Figure 7 shows the relationship between $R_{12}$ and 〈τ〉. The 2C-PSPs exhibited a similar linear relationship between $R_{12}$ and 〈τ〉 for this range of test conditions, regardless of the luminophore solvent used.

These results indicate that the type of luminophore solvent affects the luminescence lifetime and luminescence intensity of 2C-PSPs. In the next section, the effect of luminophore density in the binder on the luminescence lifetime is investigated using 2C- and 1C-PSPs.

### 4.2. Effect of Luminophore Amount Applied

The results in the previous section suggest that the difference in solvent properties affects the luminophore distribution in the porous binder layer and causes variations in the luminescence lifetime and intensity. In this section, the effect of the amount of luminophore applied on luminescence lifetime is discussed.

**Figure 8:**
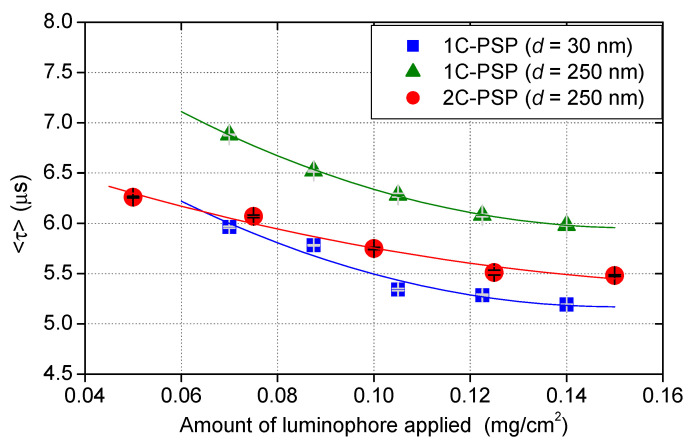
Change in 〈τ〉 with the amount of luminophore applied per sample area for 2C- and 1C-PSPs. The layer thickness was *h* ≈ 10 μm.

**Figure 9:**
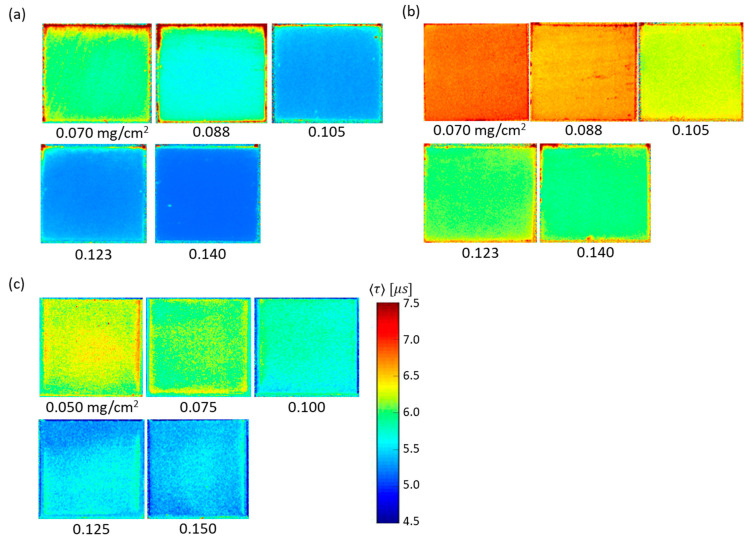
Images of the change in 〈τ〉 with the amount of luminophore applied per sample area: (**a**) 1C-PSP (*d* = 30 nm), (**b**) 1C-PSP (*d* = 250 nm), and (**c**) 2C-PSP (*d* = 250 nm). The layer thickness was *h* ≈ 10 μm.

Figure 8 and Figure 9 present the change in 〈τ〉 with the amount of luminophore applied per sample area for 2C- and 1C-PSPs. The binder thickness was *h* ≈ 10 μm. The 2C-PSPs were prepared by applying various concentrations of PtTFPP solution onto the previously applied binder layer. The 1C-PSPs were prepared by applying various amounts of PtTFPP mixed with a certain amount of binder material, as described in Section 4.1.

In both fast-PSPs, τ decreased with an increase in the amount of luminophore applied. When the same amount of luminophore was applied to a binder with TiO_2_ of *d* = 250 nm, 2C-PSPs had a shorter luminescence lifetime than 1C-PSPs. The difference in the luminescence lifetime between 1C- and 2C-PSPs can be attributed to the difference in luminophore distribution in the depth direction. The luminophores in 2C-PSPs were mainly distributed near the surface, while those in 1C-PSPs were uniformly distributed (see Figure 2). The luminescence lifetime of 1C-PSPs with *d* = 30 nm, which has a large surface area per unit mass (specific surface area), was shorter than that of 1C- and 2C-PSPs with *d* = 250 nm. The difference in the local thickness of the polymers covering the particles also affects the distribution of the luminophore. Therefore, the luminescence lifetime change in the amount of luminophore applied was more significant for the 1C-PSP than for the 2C-PSP. However, since the binder material and luminophore are applied together in the 1C-PSP, the planar variation of luminophore density is relatively small. On the other hand, 2C-PSP, in which the luminophore and binder material are applied separately, is expected to have a significant planar variation in luminophore density. In addition, the results suggest that the repair coating of fast-PSP during the test may also be a cause of the change in the luminescence lifetime distribution.

### 4.3. Effect of Thickness of the Binder Layer

**Figure 10:**
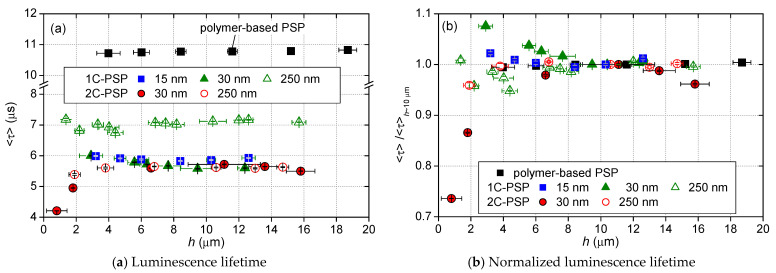
Variation in (**a**) luminescence lifetime 〈τ〉; and (**b**) 〈τ〉 normalized 〈τ〉 of 2C- and 1C-PSPs, as well as the polymer-based PSP, with binder thickness.

**Figure 11:**
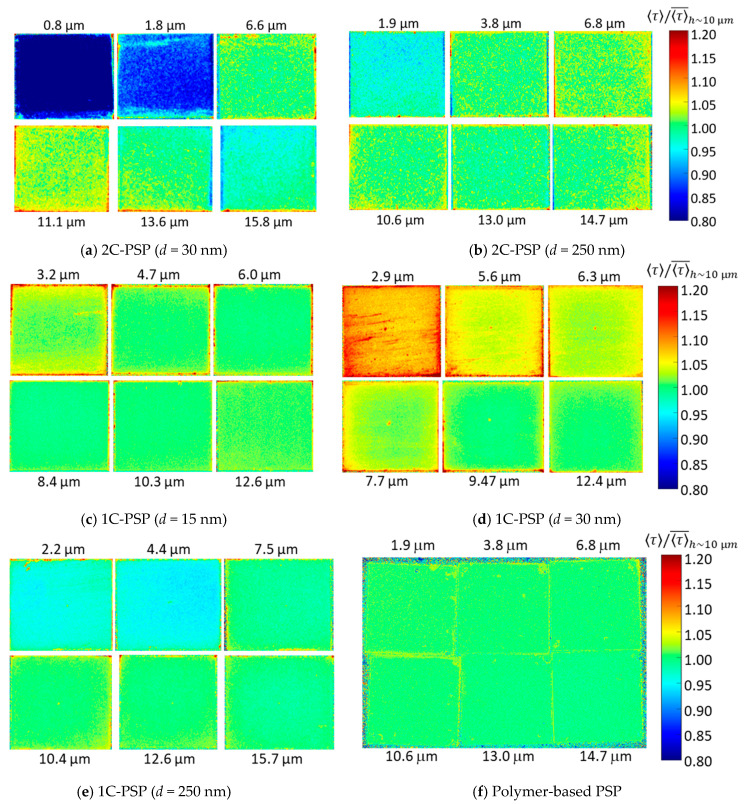
Variation in a normalized 〈τ〉 with binder thickness for 2C- and 1C-PSPs, as well as polymer-based PSP.

The effect of binder thickness on the luminescence lifetime is discussed in this section. For 2C-PSPs, a constant amount of luminophore per area of 1.0 mg/cm^2^ was applied regardless of the binder thickness. 1C-PSPs were prepared at a constant luminophore concentration of 1 mg/m for all binder thicknesses. Figure 10 and Figure 11 show the variation in 〈τ〉 for various thicknesses of 2C- and 1C-PSPs. In Figure 10b and Figure 11, 〈τ〉 was normalized by the value of 〈τ〉 at *h* ≈ 10 μm. The results of the polymer-based PSP are also presented for comparison. The polymer-based PSP showed almost a constant luminescence lifetime independent of the binder thickness because the luminophore density in the binder was constant regardless of the binder thickness. On the other hand, 2C-PSP shows a shorter luminescence lifetime at thicknesses less than 8 μm. In particular, 2C-PSPs with *d* = 30 nm at *h* = 0.8 and 1.8 μm had 26% and 13% shorter 〈τ〉 than that at *h* = 11 μm, respectively, as shown in Figure 10b. 2C-PSP with *d* = 250 nm at *h* = 0.8 μm also showed 4.1% shorter 〈τ〉. Since a certain amount of luminophore was applied to the thin binders, the luminophore density in the binder of 2C-PSP increased, resulting in shorter luminescence lifetimes.

In 1C-PSPs, the luminophore density in the binder was constant, as in polymer-based PSPs. Therefore, it was expected that the luminescence lifetime would be constant regardless of the binder thickness. However, 1C-PSPs with thinner binders showed variation in the luminescence lifetime. The reason is unclear, but it is possible that 1C-PSPs with a thickness of less than 8 μm are more likely to have variations in structure between samples. As shown above, the variation of the luminescence lifetime of 1C- and 2C-PSPs with binder thickness was slight when the binder thickness was more than 8 µm, but the variation of the luminescence lifetime was relatively large when the binder thickness was less than 8 µm.

### 4.4. Depth Variation of Luminescence Lifetime

**Figure 12:**
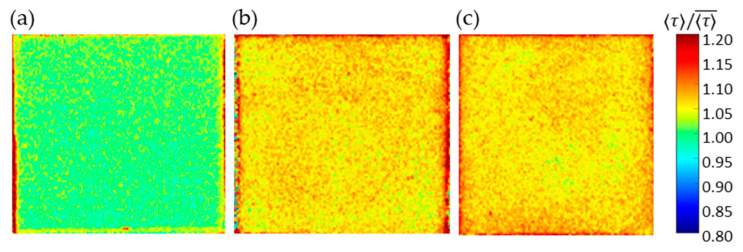
Change in normalized 〈τ〉 between the surface and the inside of the binder for 2C-PSP (*d* = 250 nm): (**a**) on surface *h* = 16.2 μm (100%), (**b**) *h* = 13.8 μm (83.8%), and (**c**) *h* = 8.5 μm (53.3%).

In this section, the depth variation of τ for 2C- and 1C-PSPs is discussed. First, 2C-PSP with *d* = 250 nm and *h* = 16.2 μm was prepared. Then, it was sanded to a thickness of 13.8 μm (83.8%) and then to 8.5 μm (53.3%) using 2000 grit lapping film (3M 261X). The change in τ between the surface and inside of the binder was compared in Figure 12 and Table 2. The lifetime τ was normalized with averaged τ at *h* = 16.2 μm before sanding. The luminescence lifetime inside the binder at *h* = 13.8 and 8.5 μm was 6.2% and 5.1% longer than that of the surface (*h* = 16.2 μm) (see Table 2). In 2C-PSP, the luminophore solution was applied onto the binder surface, which increased the luminophore density near the surface and shortened τ.

**Figure 13:**
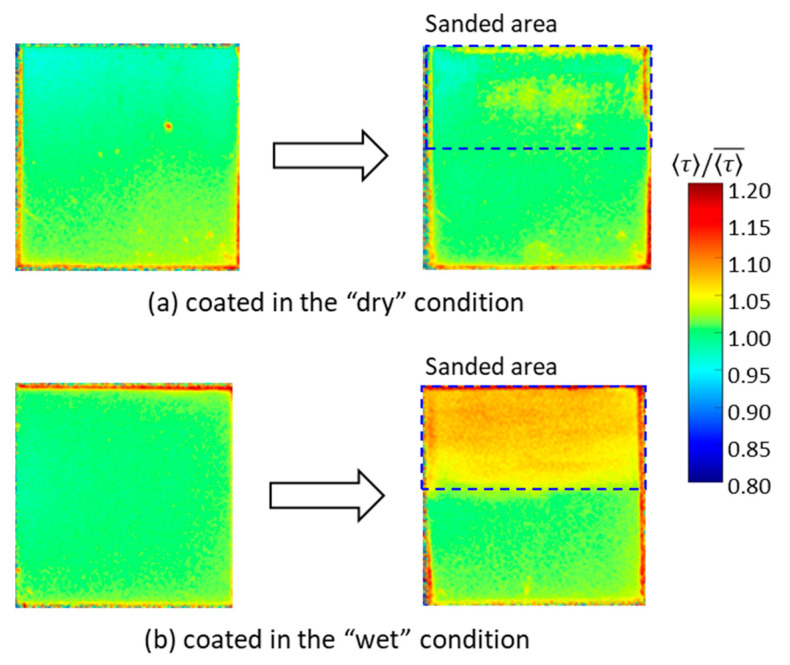
Change in normalized 〈τ〉 between the suface (left hand) and the interior (right hand) of the binder for (**a**) dry and (**b**) wet coating conditions: 1C-PSP (*d* = 30 nm and *h* = 10 μm).

Next, the change in τ of 1C-PSP in the depth direction is discussed. The 1C-PSP with *d* = 30 nm and *h* ~ 10 μm was sprayed under two different conditions: “dry” and “wet”. Under the “dry” condition, representing standard coating conditions, the spray gun was kept at a distance of about 15 cm from the sample surface to be sprayed, and the atomized PSP solution evaporated quickly on the surface. Under the “wet” condition, the spray gun was held at a distance of 10 cm, slightly closer to the sample surface, and the atomized PSP solution evaporated more slowly than in the “dry” one. Then, a part of the PSP surface was sanded with lapping paper to a thickness of about 5 μm. Figure 13 and Table 2 show the variation in τ between the surface and interior of the binder.

The sanded area showed longer τ compared to the surface. In particular, 1C-PSP coated in the “wet” condition showed a significant difference in τ between the surface and inside of the binder; the τ inside the binder in the “dry” and “wet” conditions was 2.0% and 6.2% longer than that of the surface, respectively. These results show that, even in 1C-PSP, where the luminophore and binder materials are coated simultaneously, τ can vary in depth direction depending on the coating conditions. The luminophore density may not be uniform in the depth direction because the luminophore and polymer move to the surface during the drying process. These results suggest that polishing to reduce the surface roughness may increase the τ variation of PC-PSP, especially 2C-PSPs, because the τ may vary in the depth direction.

### 4.5. Luminescence Lifetime for Large Samples

**Figure 14:**
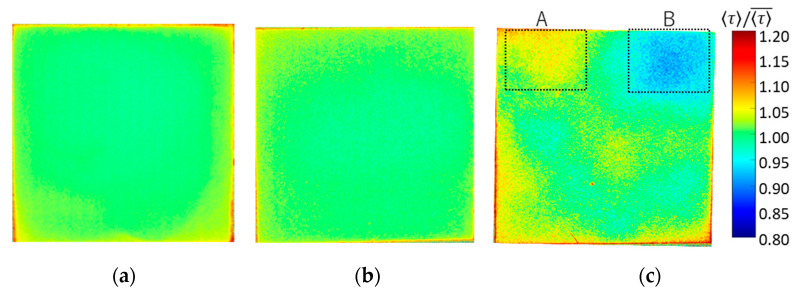
Spatial 〈τ〉 distribution of (**a**) 1C-PSP (*d* = 30 nm and *h* = 10 μm), (**b**) 2C-PSP (*d* = 250 nm and *h* = 10 μm), and (**c**) 2C-PSP (*d* = 250 nm and *h* < 6 μm).

On the basis of the above results obtained with 15 × 15 mm^2^ samples, the uniformity of the luminescence lifetime distribution was evaluated with 16-fold larger samples of 60 × 60 mm^2^. Three samples were prepared: (a) 1C-PSP with *d* = 30 nm and *h* ≈ 10 μm, (b) 2C-PSP with *d* = 250 nm and *h* ≈ 10 μm, and (c) 2C-PSP with *d* = 250 nm and *h* < 10 μm. In samples (a) and (b), fast-PSP was applied with a thickness of about 10 µm to reduce the effect of the binder thickness variation on the luminescence lifetime. On the other hand, the binder of sample (c) was coated nonuniformly in the range of 6 µm or less, where the effect of binder thickness on luminescence lifetime was significant. The top-coated luminophore solution was also applied unevenly. The spatial normalized τ  distribution of these samples (a)–(c) are shown in Figure 14 and Table 3. Samples (a) and (b) show a small variation standard deviation of 1.1% in luminescence lifetime, while sample (c) shows a larger 3.3%. In addition, the luminescence lifetime was 2.7% longer in region A and 5.8% shorter in region B of sample (c).

The variation of the luminescence lifetime was more pronounced when the binder was coated nonuniformly below 6 µm and the luminophore solution was also applied nonuniformly. Therefore, in order to obtain a uniform luminescence lifetime distribution, it is essential to coat both 1C- and 2C-PSPs as uniformly as possible, with a minimum thickness of 8 µm, as shown in Figure 10.

## 5. Conclusions

We systematically investigated the factors causing the nonuniformity of the luminescence lifetime of 2C- and 1C-PSPs. The effects of the luminophore solvents, the amount of luminophore applied, binder thickness, and spraying conditions on luminescence lifetime were evaluated.

Among the results obtained, the effect of the film thickness on the luminescence lifetime was remarkable. When the thickness of the binder was less than 8 μm, the variation in the luminescence lifetime became more pronounced. Therefore, the fast-PSP thickness should be at least 8 µm to minimize the change in luminescence lifetime due to variations in binder thickness. In addition, the luminescence lifetime of 2C-PSPs overcoated with luminophore solution on the binder changed in the depth direction. This change in luminescence lifetime is probably caused by the difference in luminophore density between the surface and the interior. The same tendency was observed in 1C-PSP when coated under “wet” conditions.

Thus, it was suggested that the nonuniformity of the planar and depth distributions of luminophores in the binder layer due to many factors, such as the amount of applied dye, differences in the volatilization rate and solubility of the dye, and the coating conditions, is closely related to the nonuniformity of the luminescence lifetime of fast-PSPs. Therefore, in order to create PSPs with a more uniform luminescence lifetime distribution, it is important to apply a fast-PSP solution so that the luminophores are uniformly distributed in the binder layer. Among the fast-PSPs tested, 1C-PSPs coated in the “dry” condition with a thickness of 8 µm or more were found to be suitable for the lifetime imaging method of PSP due to less variation of the luminescence lifetime distribution.

## Figures and Tables

**Table 1:** The normalized intensity, luminescence lifetime 〈τ〉, and pressure sensitivity at 100 kPa of 2C-PSP (*d* = 250 nm) prepared with toluene and methanol in the ratios of (a) 100:0, (b) 60:40, and (c) 20:80.

	Luminophore Solvent Toluene: Methanol	*I/I*_100:0_	〈τ〉 (µs)	Pressure Sensitivity (%/kPa)
(a)	100:0	1.00	8.02 ± 0.02	0.604
(b)	60:40	0.976	7.73 ± 0.02	0.598
(c)	20:80	0.846	5.99 ± 0.01	0.553

**Table 2:** Change in normalized 〈τ〉 in the depth direction for 2C- and 1C-PSPs.

Fast-PSP	$\langle \tau \rangle /\bar{\langle \tau \rangle }\;\mathrm{on}\;\mathrm{the}\;\mathrm{Surface}$	$\langle \tau \rangle /\bar{\langle \tau \rangle }\;\mathrm{in}\;\mathrm{the}\;\mathrm{Interior}\;$
2C-PSP (*d* = 250 nm) $\left(\bar{\langle \tau \rangle }=5.62\;\mu s\right)$	1.000 ± 0.015 (*h* = 16.1 μm)	1.062 ± 0.023 (*h* = 13.5 μm) 1.051 ± 0.023 (*h* = 8.5 μm)
1C-PSP (*d* = 30 nm) coated in dry condition $\left(\bar{\langle \tau \rangle }=5.67\;\mu s\right)$	1.000 ± 0.009 (*h* = 10.1 μm)	1.020 ± 0.013 (*h* = 5.2 μm)
1C-PSP (*d* = 30 nm) coated in wet condition $\left(\bar{\langle \tau \rangle }=5.68\;\mu s\right)$	1.000 ± 0.007 (*h* = 10.0 μm)	1.062 ± 0.012 (*h* = 5.4 μm)

**Table 3:** Normalized 〈τ〉 for 1C- and 2C-PSPs with a large sample size.

	Fast-PSP	$\langle \tau \rangle /\bar{\langle \tau \rangle }$
(a)	1C-PSP (*d* = 30 nm and *h* ≈ 10 µm)	1.000 ± 0.011
(b)	2C-PSP (*d* = 250 nm and *h* ≈ 10 µm)	1.000 ± 0.011
(c)	2C-PSP (*d* = 250 nm and *h* < 6 µm) A: (*h* = 3.0 ± 1.4 µm) B: (*h* = 2.5 ± 0.5 µm)	1.000 ± 0.033 1.027 ± 0.022 0.942 ± 0.023

## Data Availability

The data presented in this study are available on request from the corresponding author.
